# Supplementary material for: Effectiveness and Safety of Ayurvedic Medicines in Type 2 Diabetes Mellitus Management: A Systematic Review and Meta-Analysis
Source: Front Pharmacol. 2022 Jun 8;13:821810. doi: 10.3389/fphar.2022.821810 (PMC9213670; doi:10.3389/fphar.2022.821810)
Supplement: Supplementary file 1 [file DataSheet1.zip › Supplementary Material/Forest and Funnel Plots/Nigella sativa/Body weight.pdf]

Nigella sativa - Body weight

Study	Intervention N	Intervention Mean	Intervention SD	Comparator N	Comparator Mean	Comparator SD	Mean Diff. with 95% CI	Weight (%)
Heshmati 2015	36	74.80	11.30	36	77.30	14.00	-2.50 [-8.38, 3.38]	26.24
Jangjo-Borazjani 2021	20	66.36	5.26	20	71.17	6.03	-4.80 [-8.31, -1.29]	73.76
**Overall**							-4.20 [-7.21, -1.19]	

Heterogeneity: $\tau^2 = 0.00$, $I^2 = 0.00\%$, $H^2 = 1.00$

Test of $\theta_i = \theta_j$: Q(1) = 0.43, p = 0.51

Test of $\theta = 0$: z = -2.73, p = 0.01

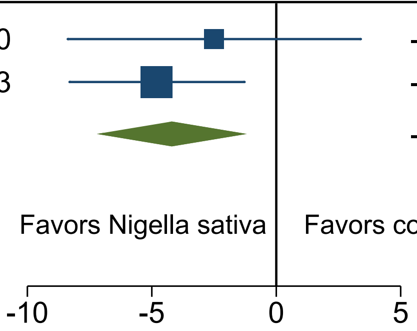

Random-effects REML model
